# Tourniquet Use in Animal Attacks: An Analysis of News Media Reports

**DOI:** 10.7759/cureus.13926

**Published:** 2021-03-16

**Authors:** Christopher M Wend, Craig Goolsby, Keke Schuler, Steven T Fischer, Matthew J Levy

**Affiliations:** 1 Emergency Medicine, George Washington University School of Medicine and Health Sciences, Washington DC, USA; 2 Department of Military & Emergency Medicine, Uniformed Services University of the Health Sciences, Bethesda, USA; 3 National Center for Disaster Medicine and Public Health, Uniformed Services University of the Health Sciences, Bethesda, USA; 4 National Center for Disaster Medicine and Public Health, The Henry M Jackson Foundation for the Advancement of Military Medicine, Bethesda, USA; 5 Emergency Medical Services, Dix Hills Volunteer Fire Department, Dix Hills, USA; 6 Department of Emergency Medicine, Johns Hopkins University School of Medicine, Baltimore, USA

**Keywords:** stop the bleed, preventable death, bystander care, hemorrhage control, tourniquet, animal attacks

## Abstract

Background

Animal attacks pose a significant public health problem in the United States. Non-venomous animals are the leading cause of mortality in these attacks, and extremity injuries leading to hemorrhage are a common pattern. The Stop the Bleed campaign advocates for public training in bleeding control tactics and public access to bleeding control kits. Controlling life-threatening bleeding, as promoted by the Stop the Bleed campaign, may be a method to reduce preventable death in these attacks.

Methodology

We searched the Nexus Uni database, which compiles international news media articles, to collect newspaper articles in the United States between 2010 and 2019 that referenced animal attacks on humans in which a tourniquet was applied. We screened articles to assess for inclusion criteria and isolated a single report for each attack.

Results

A total of 50 individual attacks met the inclusion criteria and were included for data collection. Overall, 92% (n = 46) of the victims survived the attacks, and the average victim age was 33. California was the most common location of the attacks (n = 12, 24%), sharks caused the most attacks (n = 26, 52%), and victims most often sustained isolated extremity injuries (n = 24, 48% for arm and n = 24, 48% for leg). Laypeople applied the most tourniquets (n = 29, 58%), and appliers most frequently used improvised tourniquets (n = 30, 60%).

Conclusions

While mortality in this series was low, there are hundreds of fatalities from non-venomous animal attacks each year. Equipping and training the at-risk public to stop bleeding may save additional lives. Future Stop the Bleed efforts should improve access to public hemorrhage control equipment and expand educational outreach to people engaged in high-risk activities with animals.

## Introduction

Animal attacks resulting in human injuries pose a significant public health problem in the United States. These injuries account for a large number of emergency department visits, hospitalizations, and deaths, and result in more than two billion dollars of economic losses each year [1,2]. Animal attacks caused an estimated 201 deaths annually between 2008 and 2015, and non-venomous animals were the leading cause of mortality, accounting for 57% [3]. Trauma to arms and legs with associated hemorrhage, which is common in these non-venomous animal attacks, has prompted public health interventions to minimize the sequalae of these injuries [4]. Implementing measures that improve the public’s ability to control life-threatening bleeding from animal attack injuries may be an additional method to reduce preventable deaths.

The Stop the Bleed campaign, which launched in 2015, uses lessons learned from military combat injuries to benefit the lay public via training in bleeding control tactics and access to bleeding control kits [5,6]. Stop the Bleed has reached schools, college campuses, hospitals, and other areas of large gatherings [7-9]. While intentional and mass violence is important, Stop the Bleed emphasizes the importance of bleeding control in everyday injuries [7,9-11]. Animal attacks are a unique source of injury for which Stop the Bleed interventions, such as tourniquet application, may reduce preventable death. We sought to describe the use of tourniquets in a series of attacks found in open-source, public news media. We used news media for this analysis because it serves as a collection of large population data with granular details of animal attacks and methods used to stop bleeding. Specific information such as the types of tourniquets applied and the appliers of tourniquets in these attacks does not currently exist in a more reliable peer-reviewed or government source.

## Materials and methods

Using the Nexus Uni database (LexisNexis, New York, NY, USA), which compiles international media articles, we performed a search on June 11, 2020 with the string “attack OR attacked OR bit OR bitten OR bite OR biting OR mauled AND tourniquet AND bear OR dingo OR crocodile OR dog OR wolf OR bull OR shark OR leopard OR cow OR horse OR lion OR tiger OR buffalo OR rhino OR hippo OR animal AND NOT snake.” We chose this string to isolate the most common animals involved in attacks on humans and limit non-relevant information. The search included only newspaper articles published in United States between January 1, 2010 and December 31, 2019.

After initial collection, we screened articles within Rayyan (Rayyan-QCRI, Doha, Qatar) [12] by title to exclude those that discussed topics other than animal attacks. If article titles were ambiguous, we reviewed the full text of the article and excluded those that had no mention of animal attacks. We excluded articles pertaining to clearly irrelevant topics such as sports, politics, terrorist attacks, and other human-on-human assaults. Two independent reviewers screened articles (C.W. and K.S.) and a third reviewer resolved decision conflicts (M.L.).

We performed a full article review to assess for inclusion criteria, which consisted of articles that discussed an incident in which there was tourniquet application to an arm and/or leg of a human injured by an animal. We excluded articles if the tourniquet was applied for a purpose other than bleeding control or to a non-human, if the reason for tourniquet application was other than due to an animal attack, or if the event occurred outside the United States. We also excluded articles referencing attacks that occurred outside the 2010-2019 decade. Two independent reviewers screened full texts (C.W. and K.S.) and a third reviewer resolved decision conflicts (M.L.).

Two independent reviewers (C.W. and K.S.) compared articles for story overlap to ensure that each event was only counted once. For each event, we included the article that contained the most detail without missing information contained in other articles. In one case, there was an attack with two victims as reported by multiple newspapers. We included one article that had the most information about the first victim and included another that had the most information about the second victim; thus, we treated each victim as their own event for subsequent analysis.

For each article, we collected: if the victim survived the attack as noted at the time of article publication, the year and location in which the attack occurred, the extremity or extremities injured that prompted tourniquet use, the location of other injuries, the type of tourniquet used, the role of the person who applied the tourniquet, the species of animal, and the age and sex of the injured person. We categorized tourniquet appliers as laypeople, on-duty rescuers, or off-duty rescuers. We defined rescuers as first responders and/or trained medical providers, and laypeople as anyone not meeting these criteria. Reviewers individually performed data collection (C.W., K.S., and S.F.). We gathered summary statistics using Microsoft Excel (Microsoft, Redmond, WA, USA).

If the type of tourniquet could not be determined during the review of the article, we took additional steps to find the data. Of ambiguous articles, only those referencing emergency medical services (EMS), lifeguard, or police agencies provided enough information to allow for further follow-up. For the one EMS agency referenced, we reviewed state EMS protocols to determine the type of tourniquet used. We obtained the type of one tourniquet for one police department from other public news sources. Finally, we contacted the remaining agencies via phone or email, and all of them provided the specific type of tourniquet used.

## Results

**Figure 1:**
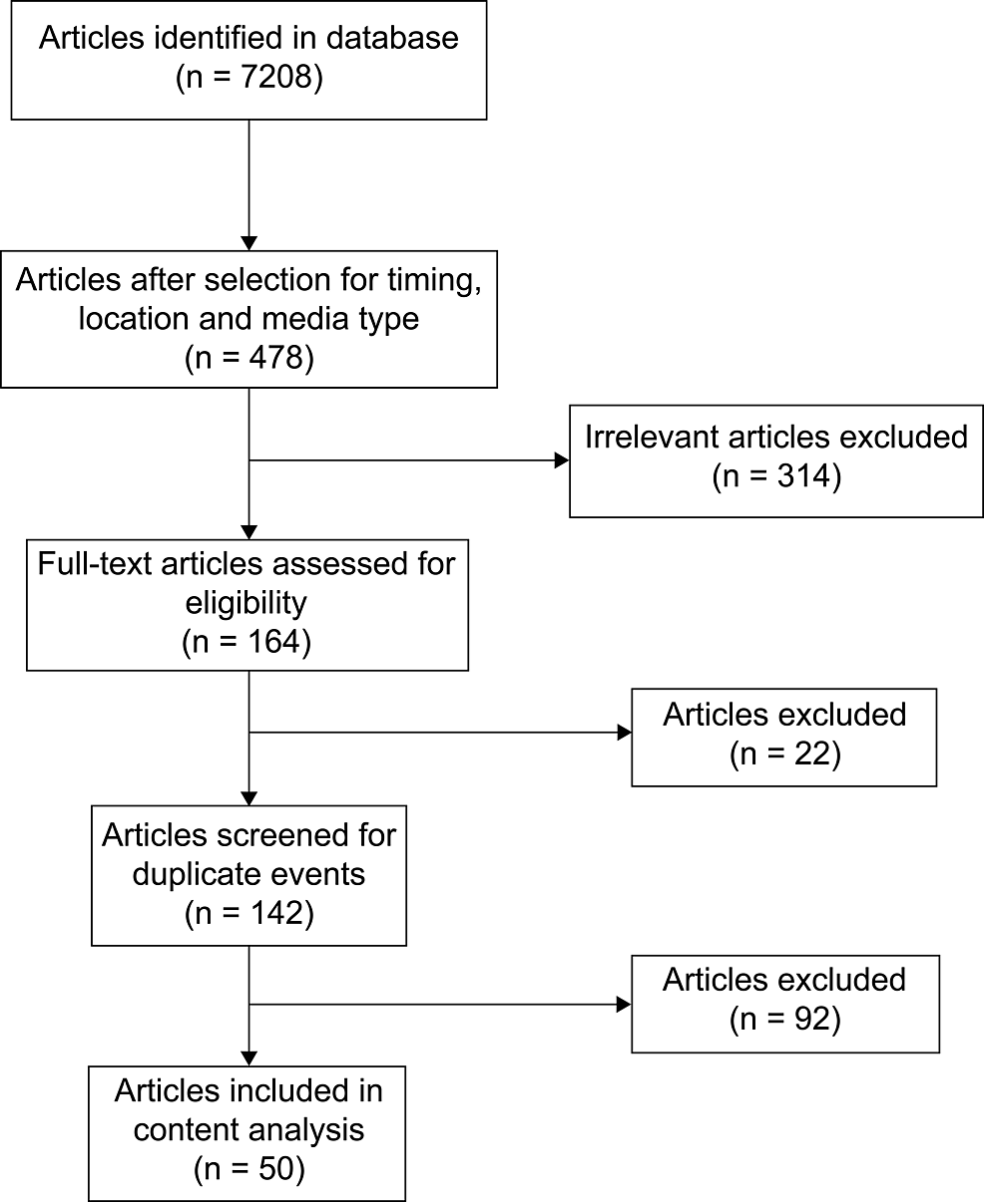
Flow diagram of article screening.

Of the 7,208 articles identified in the Nexus Uni database based upon the search string, 478 remained after selection for timing, location, and media type. We excluded 314 articles for irrelevance, and then, after full text review, excluded 22 articles that did not meet the inclusion criteria. After screening for duplicate events, we removed 92 additional articles, leaving 50 final articles for data collection. The article screening and selection process is outlined in Figure 1.

Overall, 92% (n = 46) of victims survived the attacks in this series. It is unknown if the final cause of death of the four deceased victims was related to their tourniquet application. For articles in which the data was available, the victim ages ranged from six to 62, with an average age of 33, and 66% (n = 33) of the victims were male. Attacks most commonly occurred in California (n = 12, 24%), followed by Florida (n = 8, 16%), Hawaii (n = 5, 10%), and North Carolina (n = 5, 10%) (Table 1). Victims with isolated arm or leg injuries that prompted tourniquet application comprised the majority of injuries noted (n = 24, 48% for arm and n = 24, 48% for leg), and only one victim had both arm and leg injuries prompting tourniquets. Overall, 22% (n = 11) had secondary injuries that did not result in tourniquet placement (Table 2). The most common animals that caused injuries were sharks (n = 26, 52%), dogs (n = 11, 22%), and bears (n = 6, 12%) (Table 1).

**Table 1:** Attack characteristics.

Characteristic	n	%
Victim sex		
Female	14	28%
Male	33	66%
Unknown	3	6%
Victim age (years)		
0-10	1	2%
11-20	7	14%
21-30	12	24%
31-40	6	12%
41-50	2	4%
51-60	6	12%
61-70	2	4%
Adult, but unknown age	14	28%
State of attack		
California	12	24%
Florida	8	16%
Hawaii	5	10%
North Carolina	5	10%
Alaska	4	8%
Georgia	2	4%
South Carolina	2	4%
Alabama	1	2%
Indiana	1	2%
Iowa	1	2%
Massachusetts	1	2%
Minnesota	1	2%
Mississippi	1	2%
Montana	1	2%
Oregon	1	2%
Puerto Rico	1	2%
Texas	1	2%
Virginia	1	2%
Washington	1	2%
Attacking animal		
Shark	26	52%
Dog	11	22%
Bear	6	12%
Alligator	2	4%
Beaver	1	2%
Bison	1	2%
Bull	1	2%
Cougar	1	2%
Sea Lion	1	2%

**Table 2:** Injuries sustained.

Injury	n	%
Primary injury prompting tourniquet		
Arm	24	48%
Leg	24	48%
Arm and leg	1	2%
Unknown	1	2%
Secondary injuries		
No other	36	72%
Unknown	3	6%
Yes, but specifics unknown	2	4%
Neck	2	4%
Hands	2	4%
Multiple	2	4%
Waist	1	2%
Leg	1	2%
Face	1	2%

A total of 19 articles had ambiguous tourniquet information, and 12 did not provide enough information to allow for further follow-up. However, the remaining articles collectively referenced five police departments, one EMS agency, and one lifeguard agency. In these cases, we were able to obtain the specific tourniquets used.

Appliers used improvised tourniquets in 60% (n = 30) of the attacks. Commercial tourniquets and “unknown type of tourniquet” each comprised 20% (n = 10 for each) of the attacks. Of known types of improvised tourniquets, the most commonly used items included shirts (n = 9), surfboard leashes (n = 7), and belts (n = 4) (Table 3). In three attacks, two items were used to form an improvised tourniquet and in one attack three items were used. The most common person noted to be applying the tourniquet was a layperson (n = 29, 58%) followed by an on-duty rescuer (n = 11, 22%) or off-duty rescuer (n = 10, 20%). Police officers were the most common on-duty rescuers (n = 7, 14%) and nurses were the most common off-duty rescuers (n = 3, 6%) (Table 4). With the exception of one unknown case, on-duty rescuers only applied commercial tourniquets. Additionally, of known instances, no laypeople or off-duty rescuers used commercial tourniquets.

**Table 3:** Tourniquet characteristics. *In some incidents, multiple items were used

Tourniquets used	n	%
Main tourniquet type		
Commercial	10	20%
Improvised	30	60%
Unknown	10	20%
Objects used for improvised tourniquets*		
Shirt	9	
Surfboard leash	7	
Belt	4	
Towel	3	
Dog leash	2	
Makeshift, but specifics unknown	2	
Rope	1	
String from a tent	1	
Belt from robe	1	
Rubber gloves	1	
Rubber band from spear gun	1	
Sweater	1	
Jeans	1	
Boogie board leash	1	
Sling	1	

**Table 4:** Tourniquet appliers.

Applier	n	%
Layperson	29	58%
On-duty rescuer	11	22%
On-duty police officer	7	14%
“Private safety team”	1	2%
On-duty lifeguard/emergency medical technician	1	2%
On-duty paramedic	1	2%
On-duty security guard	1	2%
Off-duty rescuer	10	20%
Off-duty nurse	3	6%
Off-duty paramedic	2	4%
Off-duty army medic	1	2%
Off-duty emergency medical technician	1	2%
Off-duty firefighter	1	2%
Off-duty medical assistant	1	2%
Off-duty police officer	1	2%

## Discussion

Animal attacks continue to be a significant cause of morbidity and mortality in the United States and remain an important public health topic [2-4]. People engaging in outdoor activities that result in interaction with wild animals and people interacting with domesticated pets remain at risk of life-threatening limb trauma. In this study, we identified 50 attacks that resulted in tourniquet placement, and four people (8%) died. In the most recent decade of Centers for Disease Control and Prevention (CDC) Wonder data available (2009-2018), 1,184 people died from interactions with non-venomous animals in the United States [13]. Similar to this CDC data, the 50 attacks in our study cohort were often by wild mammals, marine animals, reptiles, or dogs. The mortality found in the CDC data could represent a significant number of people with traumatic injuries who would benefit from education about hemorrhage control and effective equipment.

The Stop the Bleed campaign has been widely adopted throughout the United States [5], and these nationwide efforts have already begun to expand into high-risk areas of animal attacks, such as at beaches where shark attacks occur [14]. Research has demonstrated that public Stop the Bleed training can teach laypeople to effectively apply tourniquets [15], and can increase their willingness to respond to bleeding emergencies [16]. In our cohort, people not actively involved in the delivery of emergency services applied 78% of the tourniquets. These findings affirm that when life-threatening bleeding occurs after an animal attack, the people at the site of injury are the ones in a position to make the most impact and should be the target of Stop the Bleed outreach. An opportunity exists to encourage people engaged in high-risk activities, such as surfers, hikers, and hunters, to obtain Stop the Bleed training and carry their own trauma medical supplies.

In the animal attacks identified in our review, we also observed a gap between the response of laypeople and on-duty rescuers. When the tourniquet type was known, laypeople only used improvised tourniquets, while on-duty rescuers only applied commercial tourniquets. The Stop the Bleed campaign has identified the importance of using commercial tourniquets over improvised tourniquets to stop extremity hemorrhage [7,9]. Improvised tourniquets have been shown to be inferior to commercial versions in their effectiveness in stopping life-threatening hemorrhage as belts, thin wire, or other objects may generate insufficient force and directly cause tissue trauma [17-19]. In this series, appliers of improvised tourniquets often used potentially injurious objects, such as surfboard leashes, boogie board leashes, dog leashes, rubber bands, and rope. In general, improvised devices often have small widths and lack mechanical mechanisms (such as a windlass), thus risking poorer hemorrhage control effectiveness, as well as causing more severe pain and increasing the potential for damage to the underlying bodily structures. This gap in response between the public and on-duty professionals identifies a need to improve access to public bleeding control kits, with safe and proven commercial tourniquets, in high-risk areas.

Limitations

This study is limited by many factors. The source of this data is public news media which has no peer-review process nor formal fact-checking mechanism and lacks animal attacks that did not make published news. Additionally, some articles lacked or vaguely described data that we were attempting to collect, resulting in unknown answers for important datapoints. We were also limited by articles published within the Nexis Uni database which, while very extensive, may not encompass all news agencies within the United States. Finally, we did not analyze attacks without tourniquet application so we do not have data to which we can compare the characteristics of our cohort. Because of these limitations, the purpose of this study is not to serve as an all-encompassing epidemiologic survey of the use of tourniquets in animal attacks in the United States but to provoke future study and provide an alternative lens to understand the types of tourniquets used, the common appliers of tourniquets, and the situations surrounding these attacks, which is not otherwise available.

## Conclusions

Animal attacks on humans are a significant and potentially preventable cause of mortality in the United States. We identified a series of 50 attacks on humans that resulted in the placement of a tourniquet as reported by newspaper articles from 2010 through 2019. Sharks and other wild animals caused the most injuries, laypeople most commonly applied tourniquets, and appliers most often used improvised tourniquets. While 92% of those attacked survived, hundreds are known to have died each year from non-venomous animal attacks and may benefit from proper bleeding control tactics. Future expansion of Stop the Bleed efforts should include better access to public hemorrhage control supplies and educational outreach to people engaged in high-risk activities.
